# Seroepidemiololgy of rickettsioses in Sri Lanka: a patient based study

**DOI:** 10.1186/1471-2334-11-328

**Published:** 2011-11-25

**Authors:** Veranja Chathurani Liyanapathirana, Vasanthi Thevanesam

**Affiliations:** 1 Department of Microbiology, Faculty of Medicine, University of Peradeniya, Peradeniya, Sri Lanka

## Abstract

**Background:**

Rickettsioses are emerging infections in Sri Lanka as shown by the increase in the number of clinically diagnosed rickettsial patients being reported to the Epidemiology Unit, Sri Lanka. However, mapping the disease for the whole island with laboratory confirmed cases has not been previously carried out.

**Methods:**

615 samples received from 23 hospital representing 8 provinces were tested using ELISA or IFA methods and clinical data was collected using a validated questionnaire.

**Results:**

Rash was found among more spotted fever seropositive patients than scrub typhus seropositive patients while the opposite was true for the presence of eschar. Spotted fever and scrub typhus was found in a geographically restricted manner. Consistent temporal patterns were seen for the presentation of patients with rickettsioses in Kandy and Kurunegala districts for 2009 and 2010.

**Conclusions:**

This study expanded knowledge on the distribution of rickettsioses in Sri Lanka and their clinical profiles which in turn helps in the clinical diagnosis of these infections.

## Background

Scrub typhus (ST), spotted fever group rickettsioses (SFG) and typhus fever group (TFG) collectively known as rickettsioses are a group of vector borne infectious diseases caused by organisms belonging to the genera *Rickettsia *and *Orientia*. The incidence of rickettsial infections have increased worldwide in the recent past and Sri Lanka has also experienced a similar trend according to the notification data of Epidemiology Unit, Ministry of Health, Sri Lanka [1].

The notification of the disease is mainly based on clinical awareness coupled with rapid defervescence of fever in response to appropriate antibiotic therapy (macrolides or chloramphenicol) as the only laboratory test currently available for diagnosis is the Weil Felix test [1]. Two to three research centers in the country offer IFA as a means of diagnosis, but the population catered to is limited.

In addition to reducing morbidity in the local population, the recent development of eco tourism adds impetus to the need for identifying different rickettsial infections present in different regions of Sri Lanka, their reservoirs and vectors. A first step for this would be mapping rickettsial infections in patients in the different regions of Sri Lanka.

Although clinical features of rickettsial infections are generally non specific, certain patterns of clinical features could be recognized as being specific to some diseases. Collective grouping of clinical features may help identify patterns which in conjunction with epidemiological data would aid in diagnosis, especially in settings where laboratory facilities for diagnosis is minimal.

Thus, clinical profiling and mapping of the disease for Sri Lanka is a justifiable endeavor in rickettsial research in Sri Lanka. Limitations of an island wide study would include logistics of sample collection, transportation and finance. A feasible alternative to begin mapping would be to select a few sentinel sites with high levels of case reporting and using established laboratory methods, determine the pattern of rickettsioses in these areas.

The objective of the study was to map rickettsial infections in selected localities of Sri Lanka by using serological testing and to describe the clinical profiles of patients with laboratory confirmed rickettsial infections.

## Methods

Clinicians of selected hospitals were informed about the study through letters and workshops conducted in collaboration with the Epidemiology Unit and were requested to send serum samples from patients in whom a clinical diagnosis of rickettsioses was being considered according to the surveillance case definition given by the Epidemiology Unit of Sri Lanka which included fever with [2]. Paired sera taken at 10-14 day intervals were encouraged over single serum samples. Samples were stored at -20°C on arrival at the Department of Microbiology, Faculty of Medicine, University of Peradeniya and batch tested on a weekly basis. Clinical data were collected by using a validated questionnaire.

Ethical clearance was obtained from the Ethical Review Committee of the Faculty of Medicine, University of Peradeniya, Sri Lanka and informed written consent was obtained from the patients.

### Serological testing

Samples were tested using scrub typhus and spotted fever IgM and IgG ELISA kits (Panbio, Australia), IFA kits donated by the Rickettsial reference laboratory, Geelong, Australia and IFA kits prepared using antigens donated by the Rickettsial reference laboratory in Marseille, France. Patients were categorized into three groups according to the test used (Table 1).

**Table 1:** Description of the three cohorts

Patient group	Time frame	Number of samples tested	Test/tests used	Basic demographic data
Cohort 1	October 2007 to December 2008	141	Scrub typhus IgM and IgG ELISA	Age 20.7 ± 19.6 years
				
				49% Female
				
				51% Male

Cohort 2	January 2009 to December 2009	262	Scrub typhus IgM and IgG ELISA and Spotted fever IgM and IgG ELISA	Age 20.5 ± 19.9 years
				
				49% Female
				
				51% Male

Cohort 3	January 2010 to February 2011	212	IFA--screening for group specific antibodies (Scrub typhus, Spotted fever and Typhus group)--IgM and IgG*	Age 15.1 ± 19.6
				
				51% Female
				
				49% Male

*Titre determination carried out for species shown below on screen positive samples

Scrub typhus--*O.tsutsugamushi *Gilliam, Karp and Kato strains

Spotted fever--*R. honei, R. conorii, R.australis, R.siberica, R.rickettsii *and *R.akari*

Typhus group--*R.prowazekii *and *R.typhi*

### Interpretation of test results

ST and SFG IgM and IgG ELISA tests were interpreted according to the manufacturers' guidelines. A titre of over 1/128 was considered as positive for IgM and IgG using IFA. Positivity for either IgM or IgG or both was taken as being seropositive. Clinical data from patients whose serum was seropositive for both spotted fever and scrub typhus by ELISA was not considered for clinical profiling. Patients in cohort 3 were classified according to SFG and ST seropositive groups rather than according to the implicated agent for clinical profiling and epidemiological analysis.

Clinical data is given as a proportion where the denominator is the number of patients in whom the clinical data was available.

## Results

Classification of the patients according to the tests used and their basic demographic data is given in Tables 1 and 2. The 60 ST seropositive patients from cohort 1, the 33 ST seropositive patients and the 118 SFG seropositive patients from cohort 2 and the 75 ST seropositive patients and 80 SFG seropositive patients from cohort 3 were considered for the clinical profiling. Table 2 demonstrates the demographic data of scrub typhus and spotted fever seropositive patients.

**Table 2:** Demographic data of seropositive patients

Feature	Scrub typhus serology positive	Spotted fever serology positive
Mean age	15.4 ± 18.1	20.2 ± 20.3

Male	49%	49%

Commonest occupations of the adult patients	Males Farmers (48%)	Males Estate workers (45%)
	
	Females Housewife (60%)	Females Housewife (55%)

Commonest parental occupation in patients ≤ 20 years	Farming (35%)*	Estate workers (31%)*
	
	Manual labourers (12%)	Farmers (18%)

*Farming includes mostly paddy cultivation while estate workers indicated here involves work in tea and rubber plantations

In cohort 1, 36% of the ST seropositive patients had a rash while 67% had an eschar. In cohort 2, 10% of the ST seropositive patients and 87% of SFG seropositive patients had a rash while 55% of the ST seropositive patients and 4% of the SFG patients had an eschar. In cohort 3, 33% of the ST seropositive patients and 97% of SFG seropositive patients had a rash while 57% of the ST seropositive patients and only 0.01% of the SFG seropositive patients had an eschar. These differences were found to be statistically significant. Other clinical features of ST and SFG seropositive patients are mentioned in Table 3.

**Table 3:** Comparison of main clinical features in scrub typhus and spotted fever seropositive patients

Serological group	Cohort	Presence of rash	Presence of eschar	Headache	Myalgia	Arthralgia	Localized lymphadenopathy	Generalized lymphadenopathy	Hepatomegaly	Splenomegaly	Conjunctival injections
ST seropositives	1	36%	67%	88%	35%	36%	33%	02%	40%	23%	14%
	
	2	10%	55%	55%	39%	43%	20%	21%	23%	13%	00%
	
	3	33%	57%	39%	33%	22%	15%	14%	16%	10%	06%

SFG seropositives	2	87%	04%	58%	68%	67%	21%	00%	09%	09%	16%
	
	3	97%	01%	37%	53%	71%	12%	00%	06%	00%	06%

**Figure 1:**
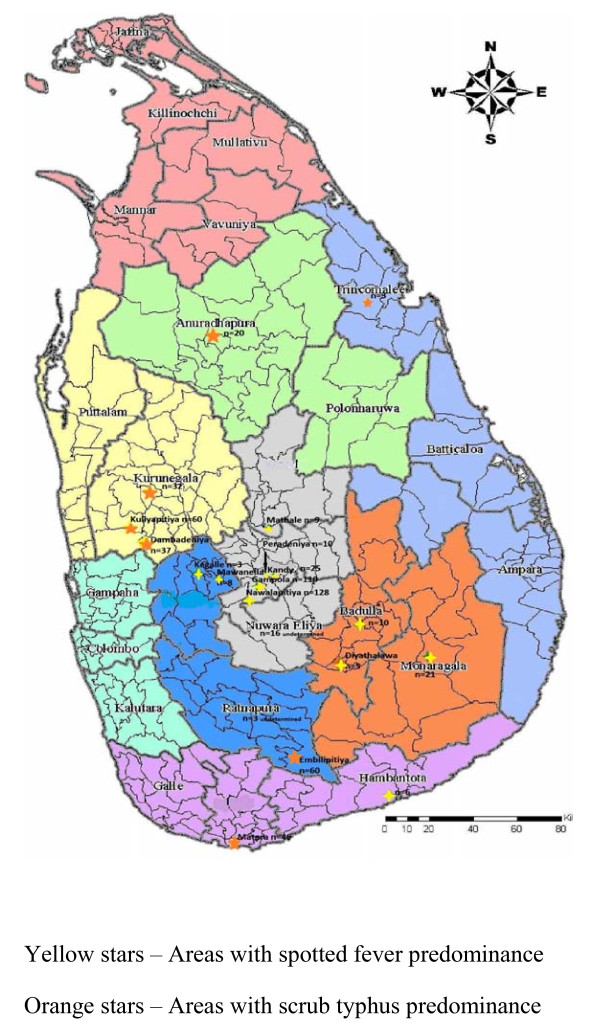
**Geographical locations of hospitals which sent ≥3 samples for the study with predominant rickettsioses**.

Samples were received from 23 hospitals representing 8 provinces of the country with the exception of the Northern Province. Number of samples received from Western and Eastern provinces was low. Seropositive patients were found in the Central, Sabaragamuwa, North Western, North Central, Uva and Southern Provinces (Figure 1).

Spotted fever rickettsioses was the predominant rickettsioses found in patients presenting to Base Hospitals (BH) Gampola, Nawalapitiya, Mawanella, Matale, Hanbantota, Diyatalawa and Monaragala, General Hospitals (GH), Kegalle and Badulla and Teaching Hospitals (TH), Kandy and Peradeniya. Scrub typhus was the main rickettsioses found in patients presenting to Base Hospitals Kuliyapitiya, Dambadeniya, General Hospitals Trincomalee, Matara and Teaching Hospitals Kurunegala and Anuradhapura.

**Figure 2:**
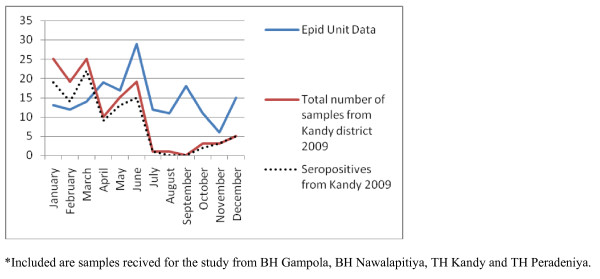
Samples received for the study from hospitals in Kandy district* compared with the notification data from Epidemiology Unit, Sri Lanka for Kandy district [1], for 2009.

**Figure 3:**
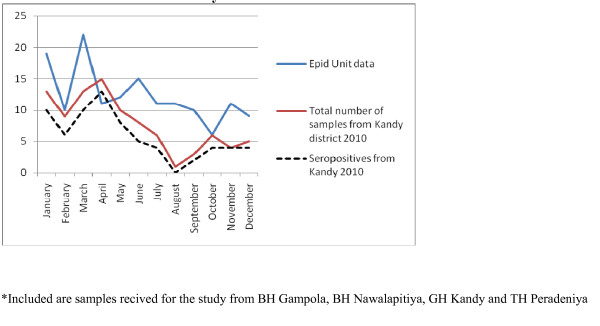
Samples received for the study from hospitals in Kandy district* compared with the notification data from Epidemiology Unit, Sri Lanka for Kandy district [1], for  2010

**Figure 4:**
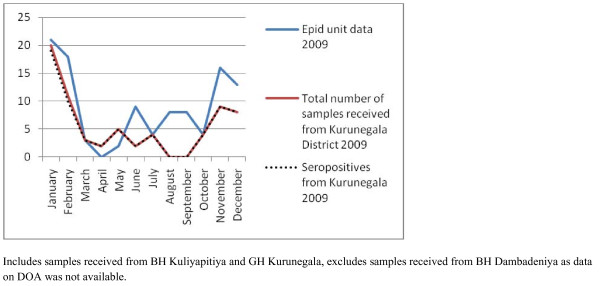
Samples received for the study from hospitals in Kurunegala district* compared with the notification data from Epidemiology Unit, Sri Lanka for Kurunegala district [1], for 2009

**Figure 5:**
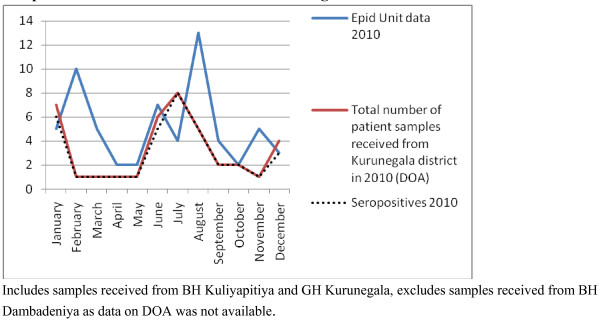
Samples received for the study from hospitals in Kurunegala district* compared with the notification data from Epidemiology Unit, Sri Lanka for Kurunegala district [1], for 2010

**Figure 6:**
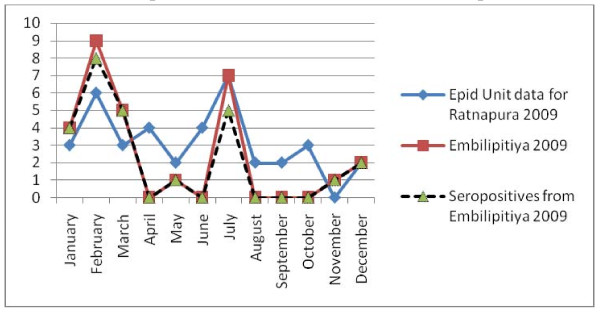
Samples received for the study from Base Hospital, Embilipitiya compared with the notification data from Epidemiology Unit, Sri Lanka for Ratnapura district [1], for 2009.

As the number of samples received from many hospitals were low, only four hospitals (Base hospitals Gampola, Nawalapitiya, Embilipitiya and Kuliyapitiya) were selected to identify the possible temporal patterns in disease occurrence. Gampola and Nawalapitiya belong to the Kandy district, Kuliyapitiya belongs to the Kurunegala District and Embilipitiya belongs to Ratnapura district. Date of admission of the patients with suspected rickettsial infections were categorized on a monthly basis and plotted for the two consecutive years of the study to see if a similar pattern would emerge. Next, the data on date of admission of samples received from all hospitals in Kandy and Kurunegala districts were plotted against the data published by the Epidemiology Unit in the Weekly Epidemiological Returns and data from BH Embilipitya was compared with the data of Ratnapura district (Figures 2, 3, 4, 5 and 6).

## Discussion

This study confirms that both SFG rickettsioses and ST are found in a geograpically restricted manner in Sri Lanka. Serological methods were used in the confirmation of clinical cases. Both serological tests were interpreted according to manufacturers' recommendations as location based seroprevelance studies have not been conducted for most areas of Sri Lanka [3].

When the epidemiological features of the seropositive groups were compared, two differences were noted. The mean age of the ST seropositive patients were less than that of the SFG. This could have been due to greater participation by paediatricians than physicians from areas endemic for scrub typhus than an actual higher incidence of disease in children. Secondly, in the SFG seropositive group, the commonest occupation of adults and commonest parental occupation in paediatric patients was work in the estate sector. In the current study, the majority of SFG samples were received from BH Nawalapitiya and BH Gampola which drain patients from the surrounding estates. For the ST seropositive group, the main occupation of adult patients and of parents of paediatric patients was farming. Both farming and working in estates involve activities that bring patients in contact with ticks and mites that act as vectors of the disease. An interesting observation, previously noted by Nanayakkara et al. (2010) is that the majority of both SFG and ST seropositive female patients were housewives [4].

Rash was detected in 10-36% of ST seropositive patients while it was present in a very high proportion (87-97%) of the SFG seropositive patients. The opposite was true for the presence of eschar as 55-67% of the ST seropositive patients had an eschar while only 1-4% of the SFG seropositive patients had an eschar. When the proportions of rash and eschar were compared between these two groups in cohort 1 and 2, the differences were statistically significant. It is noteworthy that eschar was not seen in 1/2 to 1/3 of patients seropositive for ST, this may be due to differences in detection or due to different strains of *Orientia*.

Prevalence of rash, eschar and other clinical features within SFG rickettsioses depends on the implicated organism. ST is associated in about 80% cases with an eschar [5]. Ogawa et al. described the presence of eschar in 87% of patients diagnosed with ST in Japan [6]. In 1998. Liu et al. gave an 88.5% prevalence of skin eschars in a study conducted in China from 1995-2006 [7]. The prevalence of eschar in ST seropositive patients was slightly lower and could be attributed to the difficulty in detection due to the relatively darker complexion of Sri Lankans.

Rash had been noted in varying proportions in ST. While Ogawa described a rash in 93% of the study population in Japan in 1998, a more recent study conducted from 2000-2003 in Thailand described presence of rash in only 8.9% of patients [6,8]. Similar findings had been reported from China, where Liu et al. found that rash was present in 90.4% of their study population and compared it with another study in China with a 37.2% presence of rash [7]. In the current study rash was seen in 10-36% of patients. This was in accordance with some of the above mentioned studies.

Closer to home, in a study conducted in South India, a rash was seen in 22% of patients and an eschar was noted in 4% of patients with ST [9]. While, it was obvious that in comparison with spotted fever the presence of rash was less in ST patients from Sri Lanka, the relatively lesser prevalence in comparison to studies from Thailand and China may be due to the darker complexion as mentioned above.

Headache was found in 39-88% of ST seropositive patients and 37-58% of SFG seropositive patients. Myalgia was seen in 33-39% of the ST seropositive patients and 53-68% of the SFG seropositive patients. The proportion of ST seropositive patients who had arthralgia was 22-43% while it was 67-71% for the SFG seropositive group. Other clinical features studied were hepatomegaly, splenomegaly, conjunctival injection, and localized and generalized lymphadenopathy, prevalences of which were similar to those reported from other countries [8,10-12]. As the exact causative organisms for SFG and the strain for *Orientia *were not identified, a detailed comparison was not attempted.

Clinical features of seropositive groups were not compared with those of the seronegative groups as the chance of false negativity due to early sample collection was present.

A major drawback was the non availability of certain clinical data in the accompanying request forms. In some instances it was possible to call the hospitals and get the information whereas in others it was not.

Samples were received from 23 hospitals representing 8 provinces in Sri Lanka with the exception of the Northern Province. However, the number of samples received from all hospitals was not similar. This may be due to the variation in the number of patients presenting to each hospital with suspected rickettsial disease or the interest of the healthcare staff. The hospitals that sent samples constantly and regularly were Base hospitals Gampola, Nawalapitiya, Embilipitiya, and Kuliyapitiya, and General hospital Kurunegala.

ST was the main rickettsioses found in patients presenting to Base hospitals Kuliyapitiya, Dambadeniya and General hospital Kurunegala in the North Western province, General hospital Anuradhapura in the North Central Province, Base hospital Embilipitiya in Sabaragamuwa province and General hospital Matara in the Southern province of Sri Lanka.

Of these areas, Kuliyapitiya and Dambadeniya are located in close proximity in the Kurunegala District. These two areas are predominantly agriculture based areas with intermediate rain fall. General hospital Kurunegala is the tertiary care hospital in the district capital and receives patients from the city limits and patients transferred from nearby Base hospitals including Kuliyapitiya and Dambadeniya. Thus, the finding of ST as the main rickettsioses present in patients presenting to Base hospitals Dambadeniya, Kuliyapitiya and General hospital Kurunegala indicate the widespread nature of the disease in the Kurunegala district. *Orientia tsutsugamushi *is transferred mainly by the larval stages of mites of genus *Leptotrombidium *commonly known as chiggers. Agriculture, particularly rice cultivation, forestry and oil palm and rubber plantation associated work has been shown to pose risk of acquisition of ST [13]. The climatic and weather conditions of this area situated in the intermediate dry zone of Sri Lanka would provide a favourable niche for the potential vectors to thrive. General hospital Anuradhapura, which is one of the two main tertiary care hospitals in the North Central province of Sri Lanka, provides treatment to patients from areas which are predominantly agriculture based. Anuradhapura is in the dry zone of Sri Lanka and scrub jungles are plentiful, especially in areas where the primary forest gardens are destroyed. Base hospital Embilipitiya is situated in the southern most area of the Sabaragamuwa province in the Udawalawa basin. This is a relatively dry area with plenty of scrub jungles. Matara is also in the intermediate rainfall zone of Sri Lanka situated in the Southern province. Though the number of samples received from General hospital Trincomalee of the Eastern province of Sri Lanka was small, the predominant rickettsioses found there was ST. Previous studies have shown ST is the main rickettsioses found in patients presenting to hospitals in the Western Province of Sri Lanka [14]. Nagalingam in 2006 has shown the presence of ST in Jaffna district and in a few other places in Sri Lanka, but the number of samples tested was limited [15]. Therefore, this is the first time where an Island wide representative number (with the exception of the Northern and Western Provinces) of samples were tested over a period of time and serological proof of disease established for ST.

A few studies conducted prior to the ones cited were not included in the discussion as confirmation of the diagnosis in these studies was by the Weil Felix test which is now regarded as an obsolete test as sensitivity and specificity are around 50% [16]. In addition, this test has not been validated for most rickettsial infections.

Base hospitals Nawanapitiya and Gampola were the two hospitals providing the most number of samples for the study. These 2 hospitals are situated within a 1 h distance from University of Peradeniya and about 30 min from each other in the central hills of the country. Samples received from both hospitals were predominantly SFG seropositive. TH Peradeniya situated in a neighboring are has reported SFG rickettsioses during the last few years [4,17]. The presence of SFG rickettsioses in paediatric patients presenting to Base hospital Nawalapitiya has already been reported [18]. The current study's findings were in agreement with these findings. Teaching hospital Kandy which is situated within a 10 min driving distance from Teaching hospital Peradeniya interestingly receives far less number of patients with suspected rickettsioses. Teaching hospital Kandy drains patients mainly from the eastern slope of the central hills and is the referral centre for patients transferred from the same areas. Thus, it appears that the eastern slope of central hills have a lesser number of patients with rickettsioses. General hospital Matale, situated along the eastern slopes of the central hills, also reported very few patients with SFG rickettsioses.

Base hospitals Diyatalawa and Monaragala, two hospitals in the Uva province and General hospital Badulla, the provincial general hospital in the Uva Province also showed a preponderance of SFG patients. These areas, unlike the western slope of the central hills are situated in the intermediate and dry zone of Sri Lanka. The weather tends to be mostly dry but cold.

Other hospitals have forwarded less than 3 samples and are thus not discussed.

This information would be useful to commence health educational activities in the given areas. Mapping of rickettsioses in this manner is helpful to diagnose the disease in internal travel related infection too. In depth studies could be commenced in these areas in order to identify vectors and reservoirs.

Most vector borne diseases have seasonality. This holds true for rickettsial infections as well [12]. Though Sri Lanka is a small island, it is obvious that different rickettsioses are found in different areas of the country. Thus, to establish seasonality of the disease each needs to be studied over a period of time in the endemic areas. Graphical analysis of the chronology of patient presentation in Gampola, Nawalapitiya, Kuliyapitiya and Embilipitiya hospitals demonstrates a seasonal variability. When data for 2009 and 2010 were compared in the first three hospitals, similar patterns were noted. Comparison of pooled data for Kandy, Kurunegala and Ratnapura districts with data from Epidemiology unit also show similar patterns indicating that the study received representative samples. The minor differences could be explained by the non participation by some hospitals, erratic reporting of cases to Epidemiology unit and differences in the areas of residence of patients.

Notification data and laboratory based data, as shown by the number of samples received and seropositivity show remarkable congruence. Apart from concluding that the sampling procedure in the study appears to be representative of the distribution of rickettsioses in Sri Lanka, these results also demonstrate the remarkable clinical diagnostic accuracy of this infection. The specificity of clinical features for diagnosis is relatively uncommon in infections presenting with fever and rash. Algorithms using clinical features could be used for diagnosis with laboratory confirmation as a backup for atypical presentations and further research. These data therefore need to be made available to clinicians in the given hospitals. At the same time, location based field and laboratory studies need to be conducted to assess tick activity and weather patterns in these areas simultaneously to define the risk associations and establish preventive measures.

## Competing interests

The authors declare that they have no competing interests.

## Authors' contributions

VCL contributed to the design and conduct of study including serological testing and data collection, data analysis and interpretation and writing the paper. VT conceived of the study, participated in the conduct of study, data interpretation, and writing of the paper. All authors read and approved the final manuscript.

## Authors' information

L.V.C.Liyanapathirana is a lecturer at Department of Microbiology, Faculty of Medicine, University of Peradeniya, Sri Lanka and her research interests are antibiotic resistance and emerging infectious diseases.

V. Thevanesam is the Professor of Microbiology at Department of Microbiology, Faculty of Medicine, University of Peradeniya, Sri Lanka and her research interests include rickettsial infections, melioidosis, antimicrobial activity of plant products and infection control.

## Pre-publication history

The pre-publication history for this paper can be accessed here:

http://www.biomedcentral.com/1471-2334/11/328/prepub
